# Virulence factors and mechanisms of paediatric pneumonia caused by *Enterococcus faecalis*

**DOI:** 10.1186/s13099-022-00522-z

**Published:** 2023-01-09

**Authors:** Zhiying Tian, Asif Iqbal Khan, Ata Ur Rehman, Ting Deng, Chao Ma, Liang Wang

**Affiliations:** 1 Laboratory of Biochemistry and Molecular Biology, Department of Biotechnology, College of Basic Medicine, Dalian Medical University, Dalian, China; 2 National Joint Engineering Laboratory, Regenerative Medicine Centre, Stem Cell Clinical Research Centre, The First Affiliated Hospital of Dalian Medical University, Dalian, China

**Keywords:** *Enterococcus faecalis*, Type 2 innate lymphocyte, Lipoteichoic acid, Paediatric pneumonia, Virulence factor

## Abstract

Paediatric pneumonia is a respiratory infection that affects infants and young children under the age of 3. This disease is the leading cause of infant and child mortality in developing countries because of the weak immune system of young children. The difficulty and length of time required to identify the pathogen and causative agent are the main reasons for this high mortality rate. In addition, the identification of certain causative agents is particularly important for the treatment of paediatric pneumonia. In this study, we explored the possible mechanisms by which pathogenic *Enterococcus faecalis* induced pneumonia in vivo. The potential virulence factors of bacteria isolated from the intestines of paediatric pneumonia patients were determined. Taken together, the results suggested that lysophosphatidic acid (LTA) from pathogenic *E. faecalis* decreases the expression of platelet-activating factor receptor (PAFR), which in turn disrupts the function of intestinal tight junctions (Occ and Ccldn1), leading to the entry of LE-LTA into the bloodstream because of the disruption of the intestinal barrier. Although LTA can enter circulation, it cannot directly infiltrate the lungs, which indicates that lung inflammation in mice is not caused by the direct entry of LE-LTA into the lungs. We further found that LTA activates immune cells, such as CD8 + T cells and type 2 innate lymphocytes, in vivo. Interleukin-6 and interleukin-17 can produce large amounts of inflammatory factors and thus promote the development of pneumonia. In conclusion, our findings demonstrate that the LTA of pathogenic *E. faecalis* in the intestine is a virulence factor that can cause paediatric pneumonia. This study found that intestinal bacterial virulence factors can induce immune responses in the lungs and blood. These findings could provide further insight into the mechanism of infectious diseases in the lung that are caused by bacteria in the intestine.

## Introduction

Bacteria in the intestinal tract have evolved over a long period of coexistence with humans, which has led them to not only stimulate adaptive immunity and aid in nutrient digestion and absorption, but also cause fatal diseases [1]. *Enterococcus faecalis* is a typical gram-positive coccus whose main potential virulence factors are its surface proteins (SPs), secreted lysophosphatidic acid (LTA), and extracellular products (ECPs) [2]. LTA is the most widely studied virulence factor that exists between the wall and membrane of *E. faecalis*; furthermore, LTA has many overlapping layers and induces the body to produce inflammatory factors such as interleukin (IL)-6 and IL-1β, resulting in a strong inflammatory response [3]. SP is a cell wall anchoring protein covalently bound to peptidoglycans, which are strong immunogenic molecules that disrupt the host cell's innate and adaptive immune responses. Indeed, SPs can be recognized by the C1 complex that then triggers the classical complement pathway and C3-converting enzymes, causing massive proliferation of neutrophils, macrophages, and natural killer (NK) cells in vivo and activating organism-specific immunity [4]. ECPs, also known as exotoxins, are substances secreted during the life of bacteria that promote substantial tissue damage and cause various inflammatory and injurious biological activities close to the infection site [5].

Approximately 70% of the immune cells in the body are present in the intestines, and these cells are responsible for most of the immune activation and surveillance functions [6]. Intestinal immunity has become an active part in the host defence system, supporting mucosal immunity and potentially regulating systemic immunity [7]. The mammalian gut is colonized by trillions of microorganisms that have a symbiotic relationship with their hosts. Nonetheless, the presence of large numbers of symbionts near the epithelial surface of the gut poses a great challenge to the host immune system, as it cannot initiate a deleterious inflammatory response to microbes while also maintaining its strong immune response to invading pathogens [8]. Intestinal immunity is closely associated with systemic bloodstream circulation, and numerous experimental studies have demonstrated that pathogenic substances produced by gut bacteria can cause lung diseases [9]. Lung health is affected by the important crosstalk between the gut microbiota and lungs, known as the “gut–lung axis” [6]. This means that toxins, including microbial metabolites, can affect the lungs through the bloodstream, causing inflammation in the lungs [10]. Inflammatory bowel disease (IBD) and chronic obstructive pulmonary disease (COPD) are chronic inflammatory diseases of the gastrointestinal tract and respiratory tract, respectively. The involved mucosal tissues are similar in embryology, structure, and physiology; therefore, there are similarities in the innate immune responses and the environmental risk factors associated with these diseases. Recent studies have partially described the crosstalk between the lung and the intestine and have revealed that microbial products and metabolites can be transferred from the intestine to the lungs through the blood, indicating this as a mechanism of communication [11].

Virulence factors produced by bacteria in the intestine interact with immune cells on the surface of the intestinal mucosa and activate the innate immune response of the organism through the recruitment of macrophages, NK cells, and some effector T cells. It has been shown that inflammatory lung diseases are associated with immune processes mediated by type 2 innate lymphocytes (ILC-2 s) [12]. ILCs are a recently characterized population of lymphoid innate immune cells that are heterogeneous but developmentally related and lack specific antigen receptors. ILCs develop in the bone marrow or foetal liver from common lymphoid precursors, including lymphoid progenitor cells [13]. In mice and humans, ILCs are relatively enriched in tissues with barrier functions, such as the intestine, lung, and skin, as their main biological function is to respond rapidly to environmental and inflammatory signals [14]. ILC-2 cells develop into NILC-2-like cells to help expel parasites and control infection both in vitro and in vivo [15]. ILC-2 cells also have acquired the ability to produce interleukin (IL)-17 [16]. Additionally, the regulation of adaptive immune cells mediated by ILCs is independent of IL-17a, IL-22, or IL-23 [17]. The activation of ILC-2 cells in vivo can be indirectly assessed by measuring IL-17 expression. The proinflammatory properties of IL-6 are well known and include several important physiological and anti-inflammatory functions. For example, the circulating concentration of IL-6 increases during severe inflammation, such as in cases of sepsis and acute respiratory distress syndrome [18]. Thus, the level of IL-6 can be used as an indicator to directly evaluate the extent of the inflammatory response.

Traditionally, paediatric pneumonia is thought to be caused by respiratory infections. However, insufficient understanding of the underlying causative mechanisms and limited knowledge of the causative factors have hindered the development of targeted therapies and led to antibiotic abuse [19]. Hence, a better understanding of the potential role of ILC-2 s in the intestine and lungs of paediatric pneumonia patients may help close this gap in knowledge. In this study, pathogenic *E. faecalis* was investigated as a potential pathogenic mediator of pneumonia in vivo. Notably, LTA from *E. faecalis* that was present in the intestine was found to contribute to the development of paediatric pneumonia, further demonstrating that gut and lung immunity are linked through the important hub of the blood [20]. These findings will help elucidate the pathological mechanisms of paediatric pneumonia induced by pathogenic *E. faecalis*.

## Materials and methods

### Identification of strains by 16S sequencing

After pathogenic *E. faecalis* (*LE*) and non-pathogenic *E. faecalis* (*NE*) were amplified and cultured and their potential pathogenicity was eliminated, genomic DNA extraction was performed by picking a single colony and using the Ezup Columnar Bacterial Genomic DNA Extraction Kit according to the manufacturer’s instructions (Sangon, Shanghai, China). The extracted DNA was amplified in a PCR instrument (Thermo Fisher, USA), and the amplified product was purified using the SanPrep Column DNA J Gel Recovery Kit (Sangon, Shanghai, China) [21]. Plasmid ligation was performed using the pMD®18-T Vector ligation kit (Takara, Japan), detected on a sequencer (Thermo Fisher, USA), and compared to the 16S rDNA sequences on the Ribosome Database (http://rdp.cme.msu.edu/index.jsp) [22].

### Extraction of bacterial virulence factors

*LE* isolates were collected from the intestines of young children with paediatric pneumonia and *NE* isolates were collected from the intestines of healthy young children. These strains were provided by the Microbiology Teaching and Research Department of Dalian Medical University (the collection and separation of the bacteria samples that were used was approved by the children's guardians).

- (1) For the extraction of bacterial SPs, bacteria were cultured and centrifuged. Then, bacteria were incubated with PBS (Seven Biotech, Beijing, China) containing lysozyme (2 g/mL; Vazyme, Nanjing, China) [23] for 1 h at 37 °C. After centrifugation, and the precipitates were mixed with 4 mol/mL lithium chloride solution and incubated for 12 h at 37 °C. After centrifugation, dialysis was performed in PBS at 4 °C, and freeze-drying was performed at the end of the dialysis [24].
- (2) For bacterial LTA extraction, the bacteria were then mixed with 65 °C-preheated 90% phenol solution with rapid stirring for 30 min, cooled at 4 °C, and then centrifuged [25]. The upper aqueous phase was again collected and mixed with 65 °C sterile ultrapure water 3 times. DNA, RNA hydrolase, and protease K (Solarbio, Beijing, China) were added and the samples were incubated for 45 min. Finally, the aqueous phase was placed in a dialysis bag (Solarbio, Beijing, China) for dialysis in sterile water to remove the residual phenol until the liquid and FeCl_3_ no longer produced a black precipitate. The liquid was then removed and freeze-dried [26].
- (3) For bacterial ECP extraction, sterile PBS was added to the bacterial precipitate to make a 1 × 10^6^ CFU/ml bacterial culture. High-pressure cellophane (Sorlarbio, Beijing, China) was placed on *Enterococcus* agar plate [27]. The bacterial solution was coated on the cellophane, and the plate was cultured at 37 °C for 24 h. The cellophane was then washed with sterile precooled PBS. The product was collected, and the sample centrifuged (8000 × *g*, − 4 °C) to collect all the supernatant. Any residual *E. faecalis* was killed by adding a 10 mg/ml solution of phenyllactic acid (Yuanye, Shanghai, China) [28]. Then, the product was freeze-dried.

### Experimental design and animals

Male BALB/c mice (3 weeks old, 20–22 g) with stable health and reduced experimental contingency (environmental and life factors) were purchased from Liaoning Changsheng Biotechnology Co. (Shenyang, China). All mice were housed in the specific-pathogen-free Animal Center of Dalian Medical University and were fed under specific pathogen-free conditions, with free access to sterilized pure water and food. The mice were housed at room temperature (25 °C) for at least 1 week for acclimatization before the start of the experiments. All experimental animals were kept in a healthy and active state, with normal water intake, normal food intake and no disease manifestations.

After feeding for a week, 56 mice were randomly divided into the following 7 groups consisting of 8 mice each: control untreated mice, LE-SP, LE-LTA, and LE-ECP, which were immunized with one of the virulence factors (2 mg/kg intraperitoneally, daily) of *LE*; NE-SP, NE-LTA, and NE-ECP, which were immunized with one of the virulence factors (calculated using the half lethal method, by which 2 mg/kg through a daily intraperitoneal injection is the most effective infection dose) of NE‒*E. faecalis*. NE‒*E. faecalis* isolated from the intestine of healthy children was used as an experimental control to demonstrate the specific pathogenic ability of LE-*E. faecalis* and to exclude the presence of widespread antigenicity [1]. Because this experiment is a pathogenic model, we did not include recovery treatment. The experimental treatments lasted for a total of 3 days, after which the animals were euthanized and blood, lung, and colon samples were collected.

### Haematoxylin-eosin tissue staining

The colon and lungs of mice were collected and fixed in 4% paraformaldehyde (Seven, Beijing, China). Then, the organs underwent gradient dehydration, liquid paraffin wax wrapping, and wax pourer embedding. Tissue sections were cut using a microtome (Thermo Fisher Scientific, Waltham, MA; USA), and the tissue section thickness was 3–5 µm [29]. Before staining, the tissue samples were dried in an oven (Lichen Technology, Shanghai, China) at 60 °C for 2 h. The tissue slices were dewaxed, stained with haematoxylin and eosin (Solarbio), gradient dehydrated, and sealed using neutral gum (Solarbio).

### Immunohistochemistry

Antigen repair of sectioned tissues was performed using sodium citrate (Seven) antigen repair solution. The samples were then incubated with a peroxidase suppressor (Zhongshan Jinqiao, Beijing, China) for 20 min and goat serum (Zhongshan Jinqiao) for 45 min. Next, the samples were incubated at 4 °C with a goat anti-rabbit Muc-2 antibody (1:100 dilution; Proteintech, Rosemont, USA), and biotin-labelled goat anti-mouse/rabbit IgG (Zhongshan Jinqiao) was used as a secondary antibody. The samples were the incubated with horseradish enzyme-labelled streptavidin working solution for 20 min. DAB developing solution (Zhongshan Jinqiao) was prepared to fully cover the tissues, and haematoxylin staining solution was used again for nuclear staining. Finally, gradient dehydration and sealing of the slices were performed and ImageJ software was used for quantitative analysis. A total of 56 lung and intestinal tissue sections were analysed, and the positive area was automatically calculated based on the threshold. GraphPad 8.0 was used for the analysis of variance (ANOVA).

### Enzyme-linked immunosorbent assay (ELISA)

The concentration of LTA in the serum and tissue homogenates (diluted 2–5 times) was measured using the Mouse LTA Quantification ELISA Kit (Kexing, Shanghai, China) according to the manufacturer's instructions. IL-6 and IL-17 levels were also measured using ELISA kits (Enzyme Biolabs, Jiangsu, China) according to the manufacturer’s instructions. The sensitivity of the mouse LTA quantification kit was 10 ng/L, whereas that of the IL-6 and IL-17 quantification kits ranged between 3.75 pg/ml–120 pg/ml and 15 pg/ml–400 pg/mL, respectively. GraphPad 8.0 was used for the ANOVA.

### Quantitative real-time polymerase chain reaction (RT-PCR)

Total mouse lung tissue RNA was extracted using TRIzol (TaKaRa, Japan) according to the manufacturer's instructions. The RNA quantity and quality were measured using a Nanodrop spectrophotometer (Thermo Fisher Scientific) with a 260/280 ratio of 1.8–2.0. cDNA was reverse-transcribed using the HiScriptII Q RT SuperMix qPCR kit (Vazyme, Jiangsu, China) according to the manufacturer's instructions. The RNA samples were digested by DNAase (Solarbio) and then reverse-transcribed in a total PCR volume of 20 µl. Gene expression analysis was performed on a Bioer LineGene 9660 Plus system (Bioer, Hangzhou, China) using ChameQ Universal SYBY qPCR Master Mix (Vazyme Jiangsu, China). The relative amounts of each gene were normalized to that of *ACTB* (as a housekeeping gene with stable and continuous transcription) [30] and analysed using the 2^−ΔΔCt^ method. The amplification procedure was as follows: pre-denaturation at 95 °C for 30 s, denaturation at 95 °C for 10 s, annealing at 60 °C for 30 s, and extension at 72 °C for 1 min for 40 cycles. The primer sequences are as follows, *ACTB:3′*-AACGACCCCTTCATTGAC (Tm = 52.0),CCACGACATACTCAGCAC-*5′* (Tm = 53.1),*IL-6*:3*′*-TGTGCAATGGCAATTCTGAT (Tm = 57.8), GGTACTCCAGAAGACCAGAGGA-5*′* (Tm = 59.3),*IL-10*:3*′*-CTATGCTGCCTGCTCTTACTGAC (Tm = 52.2),GAGTCGGTTAGCAGATGTTGTCCAG-5*′* (Tm = 57.9) (All chemically synthesized by Songon Biotech (Shanghai) Co.,Ltd.)

### Western blotting

Total proteins were extracted from intestinal tissues or cell lysates using RIPA buffer containing phosphatase inhibitors (Solarbio), and protein quantification was performed using the Kormas G-250 kit (Solarbio). Protein lysates were separated by 10% sodium dodecyl sulfate‒polyacrylamide gel electrophoresis (Beyotime, Beijing, China) and then transferred onto polyethylene fluoroethylene membranes (Millipore, Burlington, MA, USA). The membranes were blocked for 30 min at 25 °C with a blocking buffer consisting of 5 × fast blocking solution (Beyotime) in Tris-buffered saline and 0.1% Tween-20. The membranes were then incubated overnight at 4 °C with the primary antibody and then incubated with horseradish peroxidase-conjugated secondary antibody (Proteintech). Proteins were visualized using an electrochemiluminescence kit (Keygen Biotech, Nanjing, China) and a chemiluminescence imaging system (Bio-Rad, Hercules, CA, USA). The following primary antibodies were used: β-actin (1:2000 dilution, RRID = AB_2687938),anti-Occ (1:3000 dilution, RRID = AB_2880820), anti-CLDN1 (1:4000 dilution, RRID = AB**_**2079881), and anti-PAFH (1:3000 dilution, RRID = AB**_**2878146) (all from Proteintech). The experiments were performed in triplicate. The grey bands were calculated using ImageJ, and GraphPad 8.0 was used for ANOVA.

### Immunofluorescence

Paraffin sections of lung and colon tissue were dewaxed, rehydrated, and subjected to antigen heat retrieval in citrate buffer, followed by blocking with 10% goat serum for 30 min at 25 °C. Next, the samples were incubated with mouse antibodies against ST-2 and CD8 (RRID = AB_2264785 and AB**_**2882970, 1:100 dilution; Proteintech, USA) overnight at 4 °C. After washing 3 times with PBS, the sections were stained with the goat anti-mouse lgG(H + L),HRP conjugate fluorescent secondary antibody (RRID = AB_2264785, 1:100 dilution; Proteintech) for 1 h at room temperature. Sections were washed 3 times and stained with 4*′*,6-diamidino-2-phenylindole (Seven) for 10 min at room temperature. Images were captured using an inverted fluorescence microscope (Olympus, Shinjuku, Japan). A quantitative method was used to calculate the positive areas in the tissue using ImageJ. The total number of tissue areas was 2,217,984, using the threshold automatic calculation of positive areas. GraphPad 8.0 was used for the ANOVA.

### Infrared spectrometry

After the NE-LTA and LE-LTA were completely dried, a small amount was placed on the optical platform of an infrared spectrometer (Thermo Fisher Nicolet iS50, USA). Then, potassium bromide blanks and sample pressed tablets were prepared. The pressed potassium bromide blanks (empty potassium bromide tablets without sample) were placed in the sample holder in the sample compartment of the spectrometer. The background was measured, the spectrum was named, and the acquisition of the reference background spectrum was confirmed. After the spectrum was acquired, the sample to be measured was placed in the spectrometer, and the software performed various analytical processes on the spectrum as required. Then, the report with the spectrum in different forms was printed [31]. The spectra were compared using Origin 2018.

### Purification of LTA was measured by UV spectroscopy

The purification of lipoteichoic acid was measured by UV spectrophotometry (Nondrop 2000, Thermo, USA). First, pure water was used to zero the instrument (usually repeated three times), the sample (LTA and standard) was put into the detection arm, and then A260 nm and A280 nm wavelengths were selected for measurement. The instrument provided the value after the detection was complete [32]. By comparing with the LTA standard of *Staphylococcus aureus* (Sigma, USA), we were able to determine whether the extracted substance was lipoteichoic acid. Through continuous UV detection [33], the absorption peaks of nucleic acid and protein at A260 nm and A280 nm were detected, and the residues of nucleic acid and protein in the test substance was detected. This also allowed measurement of the characteristic absorption peak of lipoteichoic acid at A300 nm.

### LTA-SDS PAGE silver staining

The extracted and purified NE-LTA and NE-LTA were electrophoresed using a 12% SDS PAGE gel plate. The gels were fixed 50% formaldehyde and 50% acetic acid for 10 min, washed with water overnight, stained with 1% AgNO_3_ for 10 min, and washed twice with water. Developing solution (3% NaCO_3_ + 0.03% formaldehyde) was added to develop the gel until the bands were clear, and a small amount of terminating solution (5% acetic acid) was added, which allowed further processing for pictures [34]. After fixation, the gels were stained with a silver staining kit (Solarbio) and photographed in a gel imager (Thermo). The main macro amphiphiles were lipoteichoic acids (LTA), which are found in most low G + C bacteria (Firmicutes). LTA is composed of a lipid anchor linked to a chain of poly-glycerol or poly-ribitol units that is separated by a phosphate group [35].

### Statistical analysis

The experimental results presented here were repeated in triplicate. The data were statistically analysed using Prism 8.0 (GraphPad Software, San Diego, CA, USA). Multiple groups were evaluated by ANOVA. A *P value* < 0.05 was considered statistically significant. Experimental results were performed with biological replicates for statistical purposes.

## Results

### LTA from *E. faecalis* induces pathological changes in the intestine and lungs

**Fig. 1:**
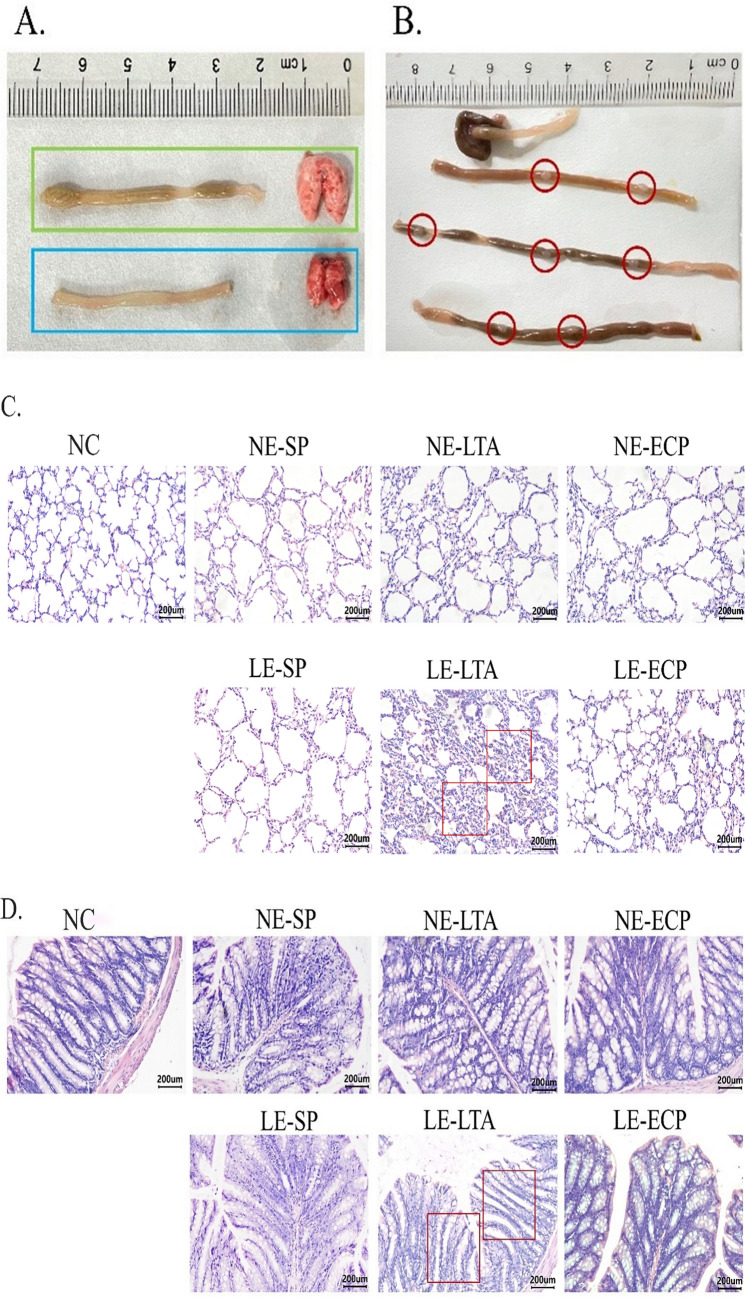
Intestinal and lung tissues collected from mice exposed to virulence factors from NE- or LE-*E. faecalis*. **A** Intestine and lungs collected from control (green) and LE-LTA-exposed (blue) mice. **B** Intestine tissues from LE-LTA-exposed mice depicting ulcerated nodules on the surface (red circles). **C**, **D** Haematoxylin-eosin staining of lung and intestine tissue samples. Red boxes indicate **C** collapsed alveoli and loss of the lung septum, as well as **D** cleaved and broken intestinal villi, with a reduced number of cupped cells. Scale bar: 200 × magnification. Abbreviations: ECP, extracellular product; LE, pathological; LTA, lipophosphatidic acid; NE, normal; SP, surface protein

First, we explored the effects that NE and LE-*E. faecalis* extracts had on the intestine and lungs of mice. Analysis of tissue samples confirmed that LE-LTA-exposed mice had significantly atrophied lungs compared with those in the control (untreated) group, and a large number of ulcerated nodules appeared on the intestines of the LE-LTA mice (Fig. 1A, B). In particular, mice in the LE-LTA group showed alveolar collapse, unclear lung septal boundaries, and a large number of neutrophil aggregates, whereas the lungs of mice in the other groups showed structures similar to those in the control group (Fig. 1C). Moreover, analysis of the colon further showed that only mice in the LE-LTA group had severe intestinal villi breakage and intestinal gland atrophy, whereas those in the other groups did not (Fig. 1D). These results suggest that LTA from LE-*E. faecalis* causes severe intestinal and pulmonary damage in mice.

### LTA from pathological *E. faecalis* disrupts the intestinal barrier

**Fig. 2:**
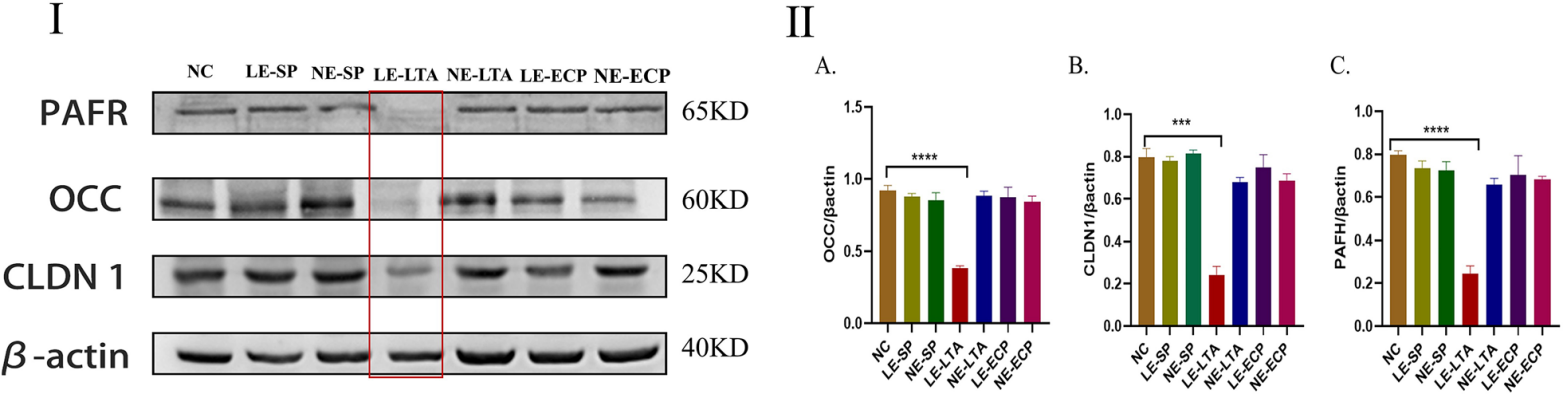
The level of tight junction proteins (Occ and Cldn1) and PAFR in the intestine (n = 8). **I** Tissue homogenates were evaluated by Western blotting. **II**-**A**–**C** Abbreviations: Cldn1, claudin-1; ECP, extracellular product; LE, pathological; LTA, lipophosphatidic acid; NC, negative control; NE, normal; Occ, occludin; PAFR, platelet-activating factor receptor; SP, surface protein. **P < 0.05

To clarify whether the inflammation in the lungs was related to the damage in the intestine, the levels of the intestinal tight junction proteins occludin (Occ) and claudin-1 (Cldn1) were evaluated by immunoblotting. The expression of Occ and Cldn1 was reduced in LE-LTA mice compared with the other groups, which all showed similar levels (Fig. 2I). These results suggest that lung injury in mice is associated with intestinal barrier disruption. Previous studies have demonstrated that decreased expression of intestinal tight junction proteins is closely related to decreased expression of intestinal platelet-activating factor receptor (PAFR) [36]. Therefore, we next evaluated whether LE-LTA exposure disrupted the intestinal barrier of mice by evaluating the amount of PAFR in the intestine. In agreement with the previous findings, intestinal PAFR levels were reduced in LE-LTA mice compared with the other groups, which all showed similar levels (Fig. 2II). Hence, the disruption of the intestinal barrier in mice exposed to LE-LTA is directly related to the decreased expression of PAFR.

### LTA from pathological *E. faecalis* decreases mucus synthesis in the lungs and intestine

**Fig. 3:**
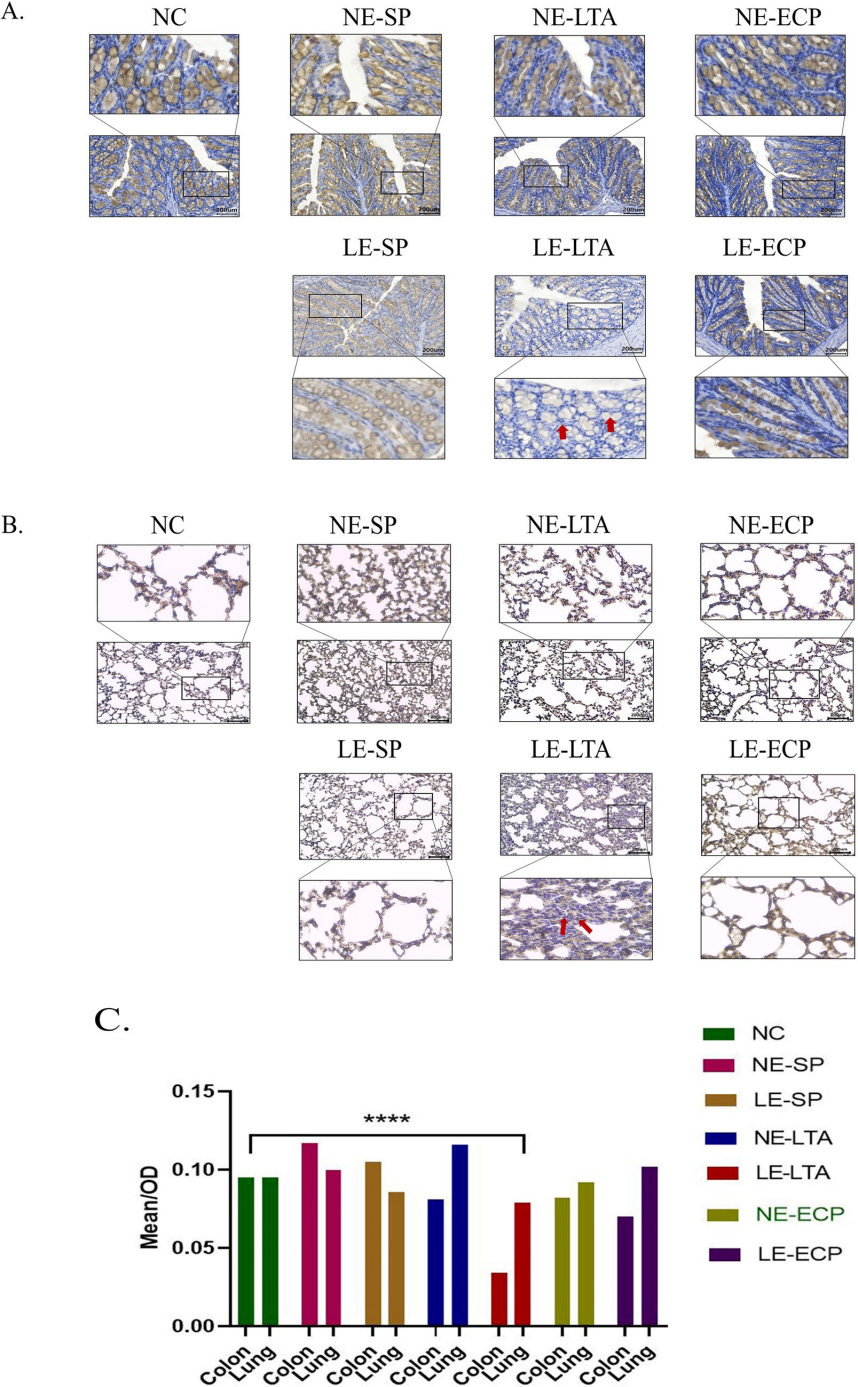
Immunohistochemical detection of Mucin-2 in the **A** intestine and **B** lungs of mice upon exposure to various virulence factors from *E. faecalis*. **C** Quantification of Mucin-2 levels in the organs using ImageJ (https://imagej.nih.gov/ij/). ***P < 0.001 for the LE-LTA group compared with the control. Abbreviations: ECP, extracellular product; LE, pathological; LTA, lipophosphatidic acid; NC, negative control; NE, normal; SP, surface protein

The lungs and intestines are the primary places where microorganisms live in the body, and their physiological structures have many similarities. For example, they both contain mechanical barriers based on tight junction proteins and mucins that specifically secrete mucus [37]. The detection of tight junction proteins and mucin in the intestines and lungs can help determine their pathological changes in models of disease. Therefore, we then explored whether virulence factors extracted from *E. faecalis* could affect mucus production. The levels of mucin 2 (Muc-2), which is a major component of mucus that is mostly secreted by cupped cells in the colon, small intestine, and respiratory tract, were evaluated by immunohistochemistry of the lungs and colon of mice. Notably, Muc-2 plays an important role in maintaining mucosal immunity by isolating microorganisms from host epithelial and immune cells. Overall, Muc-2 was significantly reduced in the intestine and lungs of LE-LTA mice compared with the control group (Fig. 3A). No significant differences were observed between the control group and the NE‒E. *faecalis*, LE-SP, and LE-ECP groups (Fig. 3B). Taken together, these results demonstrate that LTA from LE-*E. faecalis* decreases mucus synthesis in the lungs and intestine, which may increase the possibility of the direct contact of LTA with immune cells in these tissues. The ANOVA showed *P* < 0.05 (Fig. 3C).

### Immunofluorescence study on the localization of immune cells in mice

**Fig. 4:**
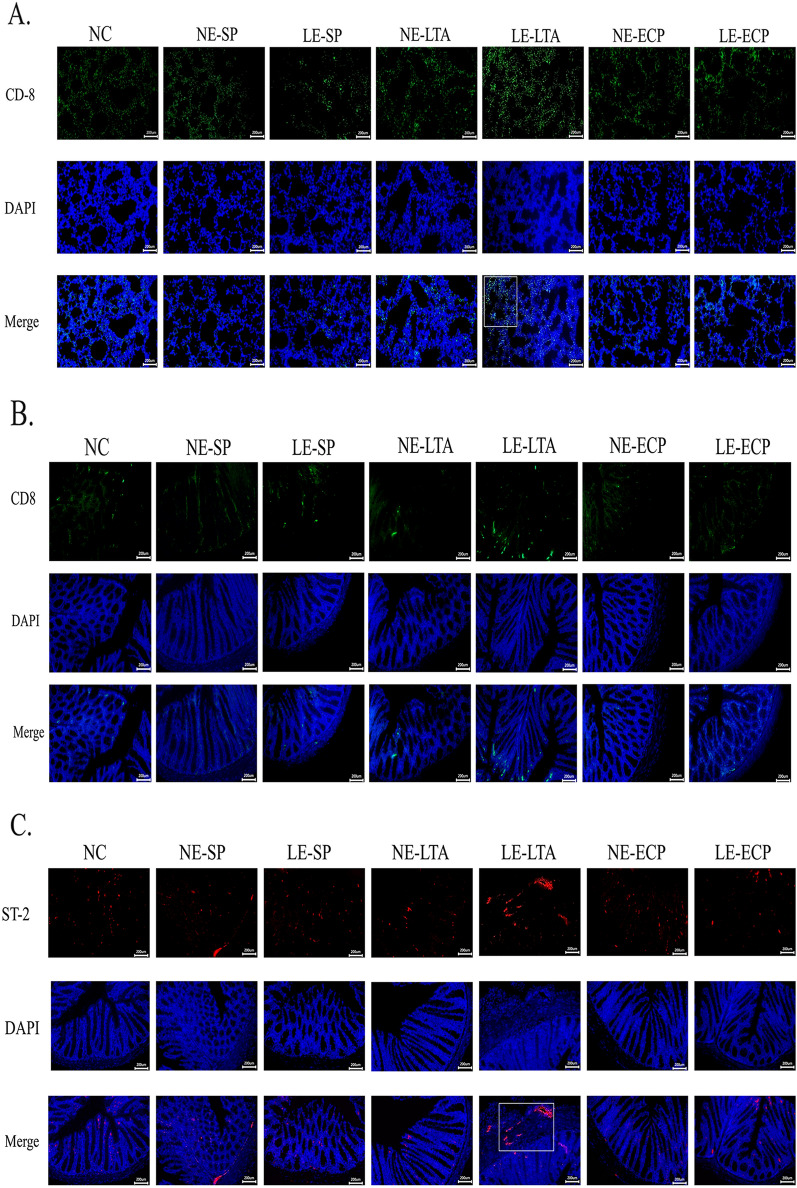

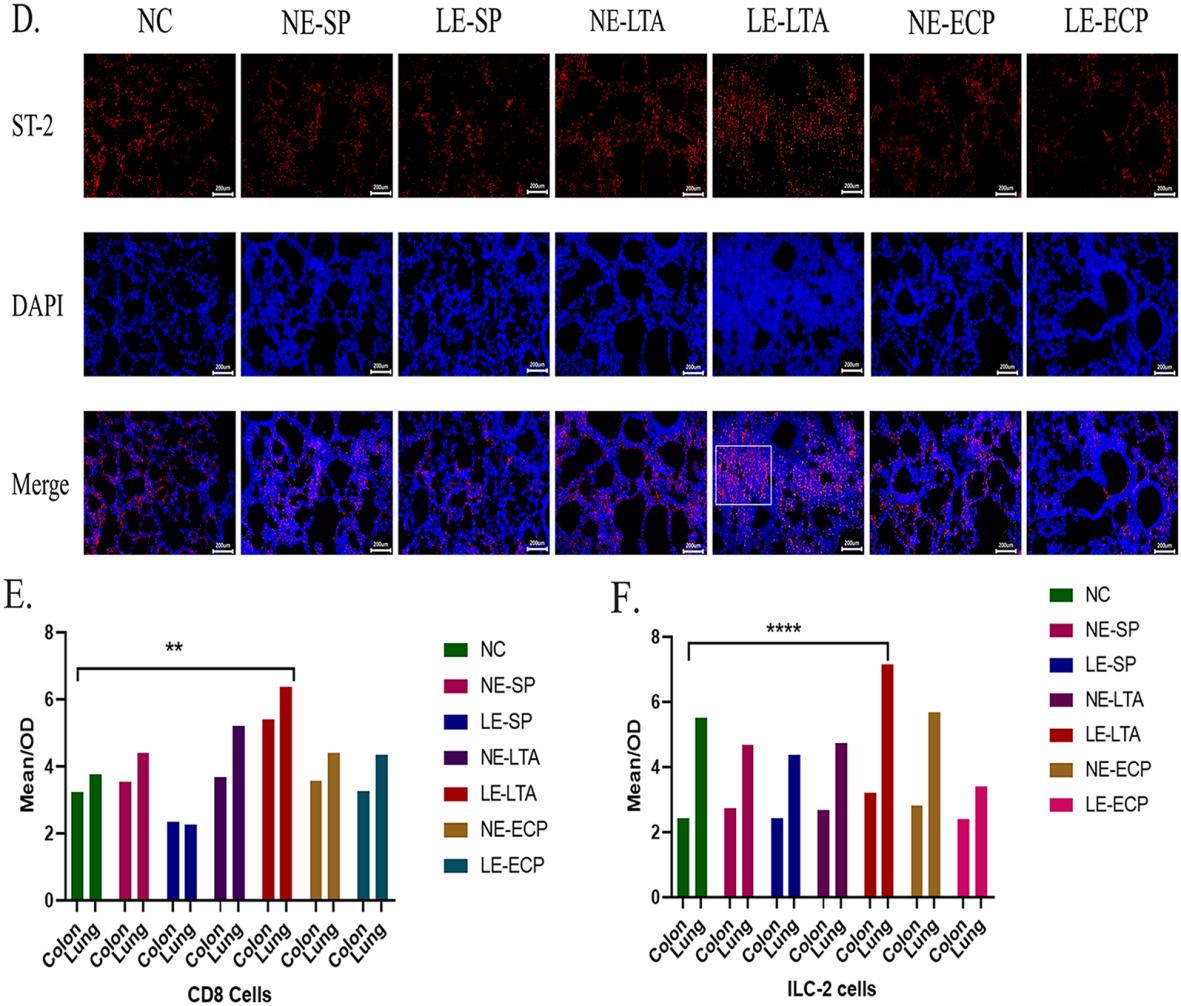
Immunofluorescence detection of ILC-2 and CD8^+^ T cells in the **A**, **C** intestine and **B, D** lungs of mice. **E**, **F** Fluorescence quantification was performed using ImageJ software (https://imagej.nih.gov/ij/). ****P < 0.001 and **P < 0.01. Abbreviations: ECP, extracellular product; ILC-2, innate lymphocyte type 2; LE, pathological; LTA, lipophosphatidic acid; NC, negative control; NE, normal; SP, surface protein

To verify whether ILC-2 s and CD8^+^ T cells facilitated interactions between the intestine and lungs through the blood, immunofluorescence was used to detect these cells in each organ. More ILC-2 s and CD8^+^ T cells were detected in the intestine and lungs of mice in the LE-LTA group than in the other groups (Fig. 4). In particular, CD8^+^ T cells showed a diffuse distribution within the lung tissue, but it was concentrated at the tips of the intestinal villi. Notably, we found that a large number of ILC-2 s were present in the alveolar collapse site and, importantly, in the myenteric vessels in LE-LTA-exposed mice. These results indicate that LE-LTA activates ILC-2 s, which in turn can travel throughout the body through the blood, with a high affinity for intestinal and lung tissues. Hence, ILC-2 s may be important mediators of pneumonia caused by LE-*E. faecalis* in the intestine.

### ELISA

**Fig. 5:**
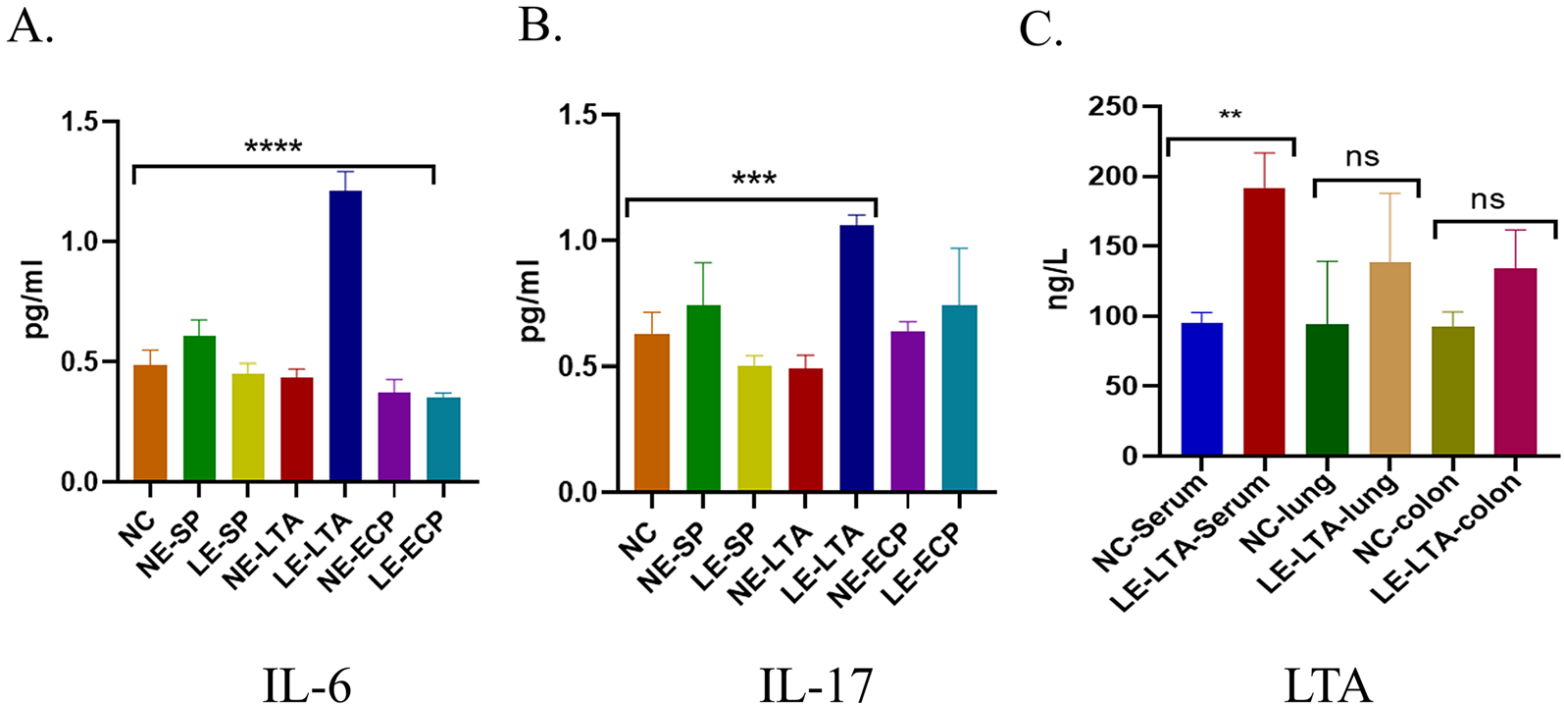
Quantification of the proinflammatory proteins (**A**) IL-6 and (**B**) IL-17 and the virulence factor (**C**) LTA in mice. **P < 0.01, ***P < 0.001, and ****P < 0.001. Abbreviations: ECP, extracellular product; IL, interleukin; ILC-2, innate lymphocyte type 2; LE, pathological; LTA, lipophosphatidic acid; NC, negative control; NE, normal; SP, surface protein

Activation of immune cells produces large amounts of active signalling molecules. Therefore, the correlation between pneumonia and the production of interleukins mediated by ILC-2 and CD8^+^ T cells was investigated. Interestingly, the relative mRNA levels of *Il-6* and *Il-17* were much higher in the lungs of LE-LTA mice than in the lungs of the other groups (Fig. 5A, B). The ANOVA showed *P* < 0.05 (Fig. 5). These results indicate that LE-*E. faecalis* causes pneumonia in mice due to the activation of ILC-2 and CD8^+^ T cells, which, in turn, produce large amounts of proinflammatory IL-17 and IL-6 in the lungs.

Finally, we evaluated the levels of LE-LTA in the serum, lungs, and intestine of exposed mice and compared them with those of the controls. The data confirmed that LE-LTA enters the circulation through the blood (*P* < 0.01), but it cannot effectively infiltrate the lungs and intestine (*P* > 0.05; Fig. 5C). Hence, LE-LTA may activate immune cells in the blood and thus mediate the development of pneumonia. In agreement with this hypothesis, the concentrations of IL-6 and IL-17 in the blood of the LE-LTA-exposed mice were found to be much higher than those of mice in the control group, whereas no significant changes were observed in the other experimental groups (Fig. 5C) *P *< 0.05. These findings were also consistent with the abovementioned increased number of CD8^+^ T and ILC-2 cells in the intestine and lungs of mice exposed to LE-LTA.

### Detection of IL-6 and IL-17 levels in mouse lungs by RT‒PCR

**Fig. 6:**
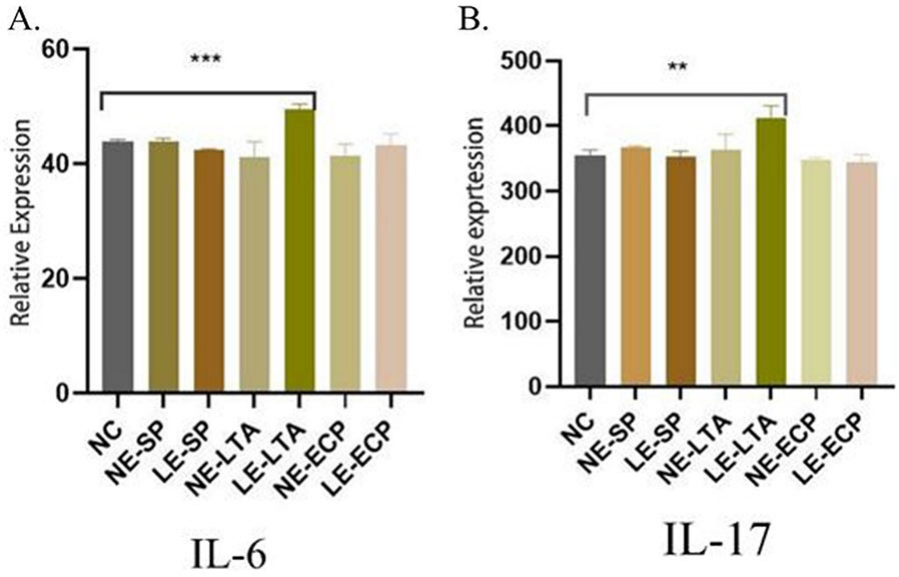
Relative mRNA expression of inflammatory factors (*IL-6* and *IL-17*) in the lungs of mice. **P < 0.01 and ***P < 0.001. As can be seen from Figure 6, IL-6 and IL-17 genes were significantly expressed in the lungs of mice in the LE-LTA group and were obviously higher than those in the untreated group NC), indicating that these two inflammatory factors were produced in large amounts in the lungs of mice in the LE-LTA group, further contributing to the development of pneumonia. Abbreviations: ECP, extracellular product; IL, interleukin; LE, pathological; LTA, lipophosphatidic acid; NC, negative control; NE, normal; SP, surface protein

We measured the expression of IL-6 and IL-17 mRNA in mice lungs. Compared with the control group, the experimental results showed that the LE-LTA group had increased expression of IL-6 and IL-17 in the lungs of mice (*P *< 0.05; Fig. 6A, B). Thus, the expression levels of IL-6 and IL-17 in the lungs and blood of mice showed a consistent upwards trend, indicating that LE-LTA can activate immune cells in the lungs and blood and cause the release of a large number of immune factors.

### Different substance structures measured by infrared spectroscopy

**Fig. 7:**
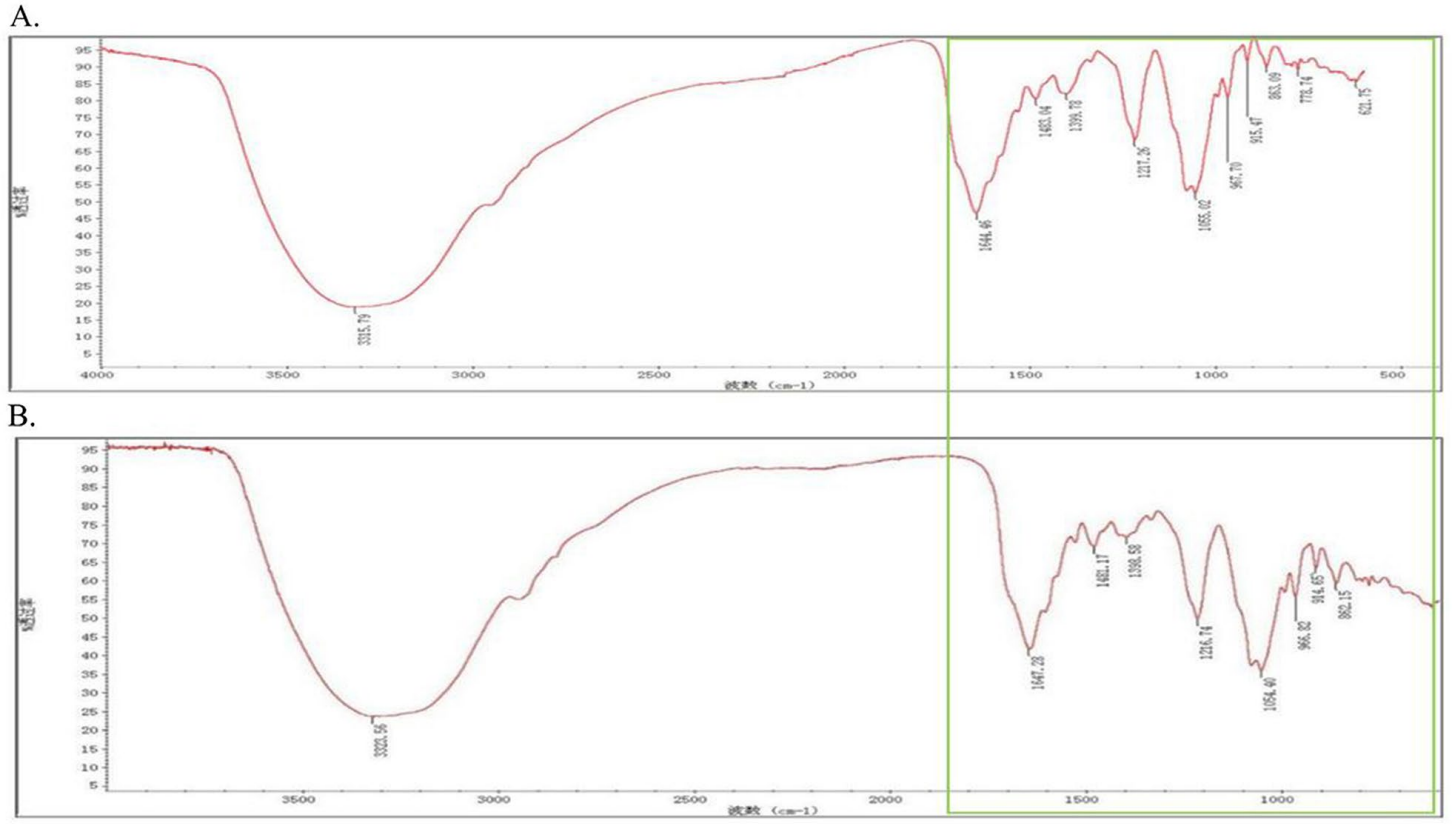
NE-LTA (**A**) and LE-LTA (**B**) have very distinct peaks within the 2000 band; their transmittance was not significantly different

The above experimental results all indicate that LE-LTA is the virulence factor of *E. faecium*, and the infrared spectrum was used to determine the structural differences of pure substances based on the different degrees of light absorption of the substances. As shown in Fig. 7, the peaks of LE-LTA and NE-LTA are different at wavelengths within 2000 nm, and this band was the decisive wavelength of different material structures. Thus, the material structures of NE-LTA and LE-LTA were distinct, and this may be related to the unique pathogenicity of LE-LTA.

### UV spectrogram of LTA detection

**Fig. 8:**
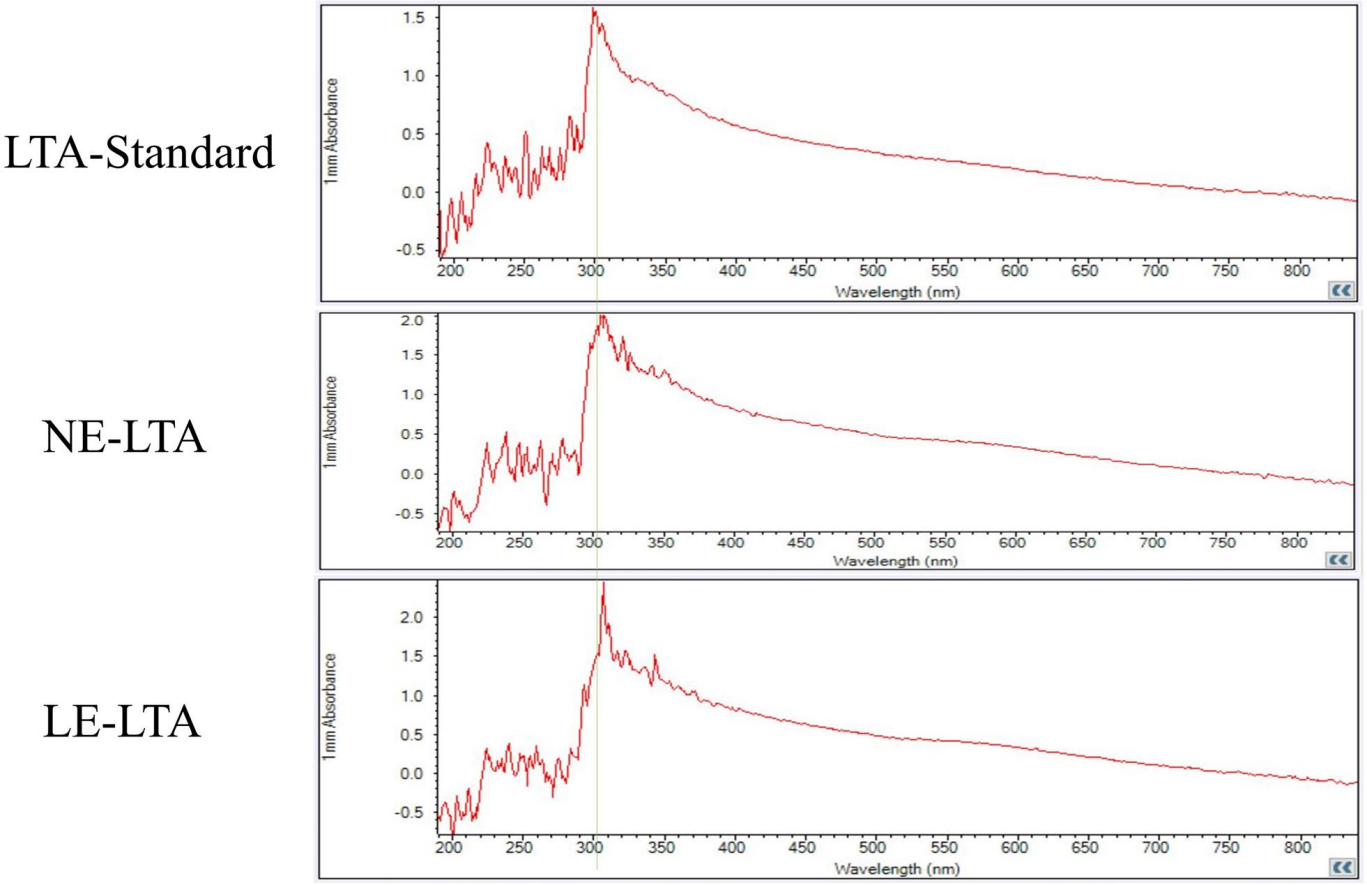
In the UV detection of LTA purity results, NE-LTA and LE-LTA had no values at A260 and A280 nm, and the standard LTA had the same absorption value at A300 nm

At A260 and A280 nm, the peaks of NE-LTA, LE-LTA and the standard are close to 0. This indicates that there is no nucleic acid or protein residue; furthermore, NE-LTA and LE-LTA have the same single peak as the standard at A300 nm (Fig. 8), indicating that the extracted LTA is a pure substance.

### SDS‒PAGE silver staining

**Fig. 9:**
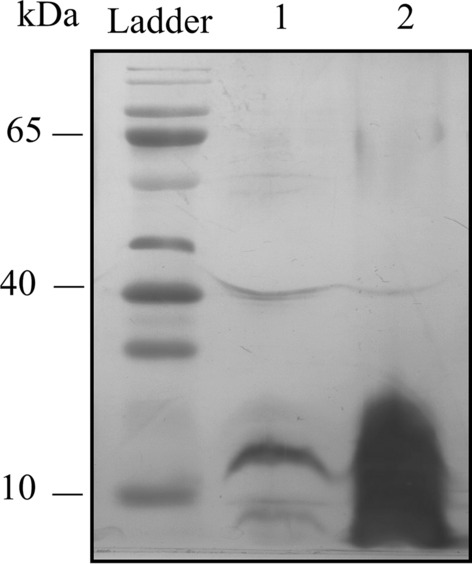
SDS‒PAGE analysis of macromolecular amphiphilic polysaccharide of *E. faecalis*. Lane 1, non-pathogenic *E. faecalis* lipoteichoic acid (NE-LTA); Lane 2, pathogenic lipoteichoic acid (LE-LTA shown by periodic acidic silver nitrate staining

SDS‒PAGE silver staining analysis revealed the apparent molecular weights of NE-LTA and LE-LTA (Fig. 9) migration modes (as broadband) to be approximately 20 kDa.

### 16S sequencing

**Fig. 10:**
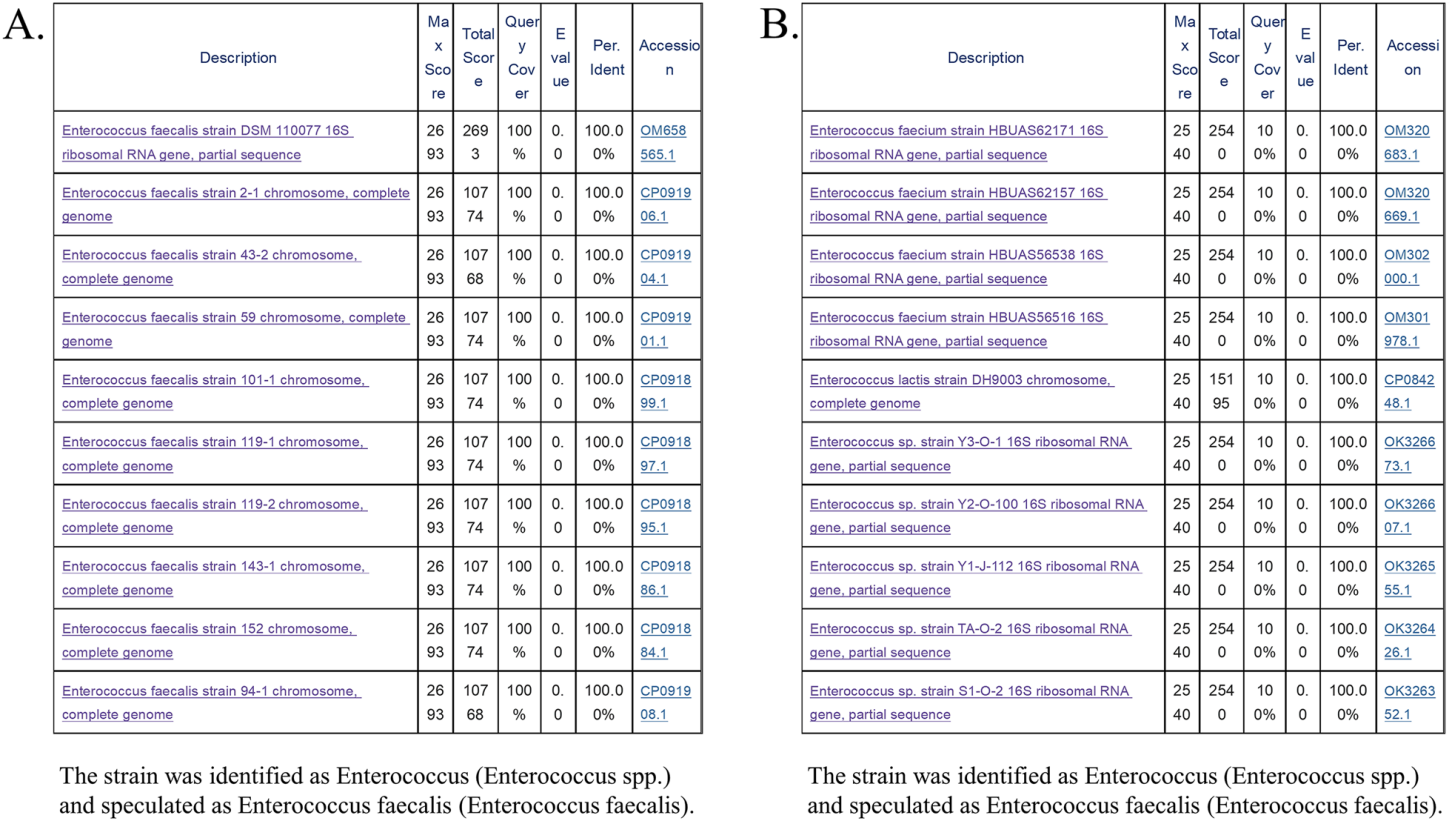
**A** Detection sequence results of NE‒E. **B** Detection sequence results of LE-*E*

In Fig. 10, after 16S full-length sequencing, it was determined that NE and LE belonged to the same genus, and they were identified as *Enterococcus* and *Enterococcus faecalis*, respectively.

## Discussion

In past studies, paediatric pneumonia has been considered to be an infectious disease of the respiratory system. However, our data demonstrate for the first time that pathogenic *E. faecalis* (isolated from the intestines of paediatric pneumonia patients) can trigger proinflammatory immune responses in the lungs, thus potentially representing an important element of the lung–gut axis. Moreover, we identified LTA as the most likely virulence factor of this bacterium that gives it the ability to cause pneumonia.

The intestines are the largest part of the immune system; they are constantly exposed to antigens and immunomodulators from the diet and commensal microbiota, but they also represent a port of entry for many clinically important pathogens. Increasing evidence also suggests that gut immune processes are also associated with the prevention of diseases affecting other body parts [38]. Based on this premise, we used changes in the gut bacteria of paediatric pneumonia patients as a starting point to uncover the aetiology of paediatric pneumonia.

As an important opportunistic pathogen that coexists with humans, *E. faecalis* has been associated with the formation of biofilms and with oral diseases, including recurrent root canal failure [39]. It is also the most common nosocomial infectious bacterium, causing endocarditis, urinary tract infections, wounds, surgical site infections, and medical device-related infections, which often become chronic after biofilm formation [40]. In vivo studies have demonstrated that LTA has strong inflammatory properties, similar to those of lipopolysaccharides, and that target-bound LTA can interact with circulating antibodies and activate complement cascade reactions, thus inducing a passive immune killing phenomenon. LTA also triggers the release of reactive oxygen and nitrogen species, acid hydrolases, highly cationic proteases, bactericidal cationic peptides, growth factors, and cytotoxic cytokines from neutrophils and macrophages, which can act synergistically to amplify cellular injury [41]. It has also been shown that massive cellular damage can lead to the development of systemic inflammatory response syndrome by producing a microbial pathogen-associated molecular pattern that activates natural immune cells through pattern recognition receptors [42]. Moreover, inflammation was shown to lead to a rapid accumulation of neutrophils, resulting in significant inflammatory lung injury [43]. These inflammatory injury responses and natural immune cell activation events are consistent with the findings of this study in the blood, intestinal tissues, and lung tissues.

The most important component of the intestinal barrier is the intestinal mucosa, which not only resists the penetration of the intestinal contents, but also importantly prevents the invasion of exogenous antigens. The intestinal mucosa serves as both a conduit for the uptake of food-derived nutrients and microbiome-derived metabolites, and as a barrier against microbial invasion into tissues and to inhibit inflammatory responses to the bulk contents of the lumen [44]. An increasing number of studies have shown that disruption of the intestinal mucosal barrier is associated with various diseases. For example, increased intestinal permeability has been observed in patients and mouse models of cardiovascular disease, and this increased intestinal permeability enhances systemic inflammation, alters intestinal immune function, and has been shown to predict adverse cardiovascular disease [45]. The gut microbiota, which is part of the brain–gut axis, may play a significant role in the connection between the common pathological mechanisms of multiple sclerosis and gastrointestinal disorders, in which gut barrier disruption has been associated with central nervous system demyelination and with gut microbiota entering circulation, ultimately affecting central nervous system microglial function [46].

Numerous studies have shown that the mucosal barrier plays an important role in maintaining the stability of the intestinal environment [47]. This physical barrier consists mainly of tightly connected intestinal monolayer epithelial cells, including stem cells, enterocytes, cupped cells, and Paneth cells, which in turn will maintain the balance between nutrient absorption and intestinal immunity by secreting mucus and blocking cellular gaps [48]. In particular, PAFR was shown to play a key role in the regulation of the pulmonary infection response in diseases such as COPD and asthma [49]. PAFR was evaluated in dextran sodium sulfate exposed mice and anti-CD40 colitis mice, and the results showed that mice in both IBD models had increased levels of neutrophils and a downwards trend in PAFR expression (50). In this study, we examined 2 intestinal tight junction proteins (Cldn1 and Occ), as well as PAFR and mucus secretion-related mucin-2. Overall, mice exposed to LE-LTA showed decreased expression of tight junction proteins, PAFR, and mucin-2, while also showing pathological manifestations of lung inflammation. Hence, the pathogenic mechanisms observed herein were very similar to those previously described, suggesting that LE-LTA leads to the disruption of the intestinal barrier, which in turn leads to the development of pneumonia in mice.

Several studies have shown that CD8^+^ T lymphocytes have specific functions in response to antigenic stimuli [51]. Concerning infections and tumours, CD8^+^ T cells are converted into cytotoxic T cells that specifically kill target cells; hence, cytotoxic CD8^+^ T cells play a key role in eliminating intracellular infections and malignant cells, and they can provide long-term protective immunity [52]. Recent studies have also shown that CD8^+^ T cells are important for the maintenance of immune homeostasis in lung tissue through memory function, and that the occurrence of lower respiratory sensitization in immunocompromised children is associated with high CD8^+^ T-cell expression. Interestingly, lung antigen-specific dysfunction was found in primary and secondary bacterial infections that produced CD8^+^ T cells with longer-lasting memory recall responses [53]. Indeed, neutralization of IL-6 with monoclonal antibodies or mice lacking mature IL-6, MyD88, or IL-6 receptors showed impaired recovery of the CD8^+^ T-cell subset [54]. These findings suggest a positive correlation between IL-6 expression and the number of CD8^+^ T cells. Recently, numerous studies have demonstrated that respiratory inflammatory diseases are associated with the expression of ILC-2 s, which promote leukocyte chemotaxis through the production of IL-17, further activating innate immune processes to promote respiratory inflammatory responses [55]. In our experiments, we measured the expression of IL-6 and IL-17 in the lungs of mice and the distribution of ILC-2 s and CD8^+^ T cells in the intestine and lungs. The results showed that high expression of ILC-2 s and CD8^+^ T cells in the intestine and lungs of mice in the LE-LTA group was accompanied by an increase in the expression of IL-6 and IL-17 in the lungs. This finding indicates that LE-LTA increases immune cell reactivity in mouse intestinal and lung tissues and produces large amounts of ILs to promote lung inflammation. The results of the infrared spectrum preliminarily showed differences in the LTA structure between *NE* and *LE* bacteria, which may be related to the specific pathogenicity of LE-LTA; however, it remains unclear why this difference leads to a change in virulence. Therefore, we hypothesize that in children with intestinal pneumonia, LE-LTA enters the blood and leads to intestinal barrier damage, leading to immune cell activation and immune factor recruitment, resulting in lung inflammation. Future studies should clarify the binding mechanism between LE-LTA and PAFR, and work to address the questions of why LE-LTA only attacks lung tissue and why LE-LTA activates ILC-2 cells. These questions still need to be addressed in the future.

In summary, this study demonstrates that (1) LE-LTA causes intestinal gland atrophy, reduced cupped cells, collapsed alveoli, blurred lung septa in mice, and severely damaging pathological changes in both lung and intestinal tissues; (2) LTA from pathogenic *E. faecalis* can disrupt intestinal tight junction protein blockade and decrease the expression of intestinal mucin, resulting in serious damage to the intestinal barrier in mice; (3) LE-LTA promotes the distribution of CD8^+^ T cells and ILC-2 s in the intestine and lungs of mice, which further increases the expression of IL-6 and IL-17 in the blood and lungs; (4) LE-LTA can break the intestinal barrier and enter the blood circulation but not the lungs; and (5) the difference in pathogenicity between *NE* and *LE* lies in the various configurations of the LTA. These data demonstrate that the virulence factor of pathogenic *E. faecalis* is LTA. We believe that understanding the immune association between the intestines and lungs will not only be helpful for the treatment of paediatric pneumonia, but it will also advance the knowledge of infection and cellular immunity.

## Data Availability

The data that support the findings of this study are available from the corresponding author upon reasonable request.

## Abbreviations

SP: Surface protein
CLDN1: Claudin-1
ECP: Extracellular product
ILC: Innate lymphocyte
LTA: Lipoteichoic acid
NE: Normal
NK: Natural killer
Occ: Occludin
PAFR: Platelet-activating factor receptor
PBS: Phosphate-buffered saline
PE: Pathogenic
